# Analysis of 1,25-dihydroxyvitamin D genomic action in human enteroids and colonoids reveals multiple regulatory effects of vitamin D in human intestinal physiology

**DOI:** 10.3389/fendo.2025.1538463

**Published:** 2025-06-04

**Authors:** Zachary K. Criss, Kali Deans-Fielder, James C. Fleet, Sylvia Christakos, Noah Shroyer

**Affiliations:** ^1^ Department of Medicine Section of Gastroenterology and Hepatology, Baylor College of Medicine, Houston, TX, United States; ^2^ Department of Nutrition Sciences, The University of Texas, Austin, TX, United States; ^3^ Department of Microbiology, Biochemistry and Molecular Genetics, Rutgers-New Jersey Medical School, Newark, NJ, United States

**Keywords:** vitamin D, gene regulation, transcriptome, small intestine, colon, organoid

## Abstract

**Introduction:**

The intestine has molecular and functional diversity across the proximal-distal and the crypt-villus axes, so it is imperative to determine the common and compartment-specific molecular actions of vitamin D. However, very little work on vitamin D mediated gene regulation has been done in normal human intestine. Here, we examined the impact of 1,25-dihydroxyvitamin D (1,25(OH)_2_D_3_) on cultures of human intestinal epithelium derived from duodenum (Dd) and distal colon (Co) biopsies of 6 subjects per tissue.

**Methods:**

Human enteroids and colonoids were cultured for 3 days to promote a stem cell phenotype (undifferentiated, Un) or to induce differentiation (Diff) and then treated with vehicle control or 1,25(OH)_2_D_3_ (100 nM). 24h following treatment enteroids/colonoids were collected, RNA was isolated and RNA-seq was performed using paired-end Illumina sequencing (analysis in R using DESeq2).

**Results and discussion:**

RNA-seq analysis showed that VDR mRNA is present in all four cultures tested (DdUn, DdDiff, CoUn, CoDiff) and it is not altered by 1,25(OH)_2_D_3_ treatment, intestinal segment, or differentiation status. 1,25(OH)_2_D_3_ induced the classic intestinal target genes TRPV6, ATP2B1 and CYP24A1 in all four culture groups while S100G was induced only in DdDiff. While 63 genes were vitamin D regulated across all four cultures (55 up, 8 down), we found that vitamin D regulated subgroups of genes within Dd, Co, Un, or Diff groups as well as set of genes that were unique to each culture. Functional analysis revealed several vitamin D-enriched gene ontologies or pathways including those for xenobiotic/drug metabolism in all four cultures. In differentiated cultures vitamin D induced genes were enriched for functions like regulation of barrier function through regulation of Rho GTPases and metabolism of lipids while vitamin D downregulated genes in Un groups were enriched for activities like water transport. These results provide new insight into 1,25(OH)_2_D_3_ genomic action in the functionally distinct compartments and segments of human intestine and suggest multiple regulatory effects of vitamin D in human intestinal physiology.

## 1 Introduction

The active form of vitamin D, 1,25-dihydroxyvitamin D_3_ (1,25(OH)_2_D_3_) is an important regulator of intestinal biology (1, 2). The action of 1,25(OH)_2_D_3_ is mediated through its binding to the vitamin D receptor (VDR), a ligand activated transcription factor that is highly expressed in the intestine (3) and that regulates gene expression by binding to vitamin D response elements (VDREs) in the promoter and/or enhancers of target genes (4). During growth, the primary role of 1,25(OH)_2_D_3_ is to regulate calcium homeostasis by increasing calcium absorption from the proximal small intestine (5). It does so by inducing genes encoding proteins that mediate apical membrane uptake (transient receptor potential cation channel V family member 6, TRPV6), intracellular calcium binding (calbindin-D_9k_, S100G) and extrusion of calcium (plasma membrane calcium ATPase, ATP2B1) (6).

In addition to the calcium-regulating function of 1,25(OH)_2_D_3_, several other intestinal functions have also been attributed to the hormone. These functions include suppression of inflammation and protection against colitis, regulation of tight junctions to control intestinal barrier function, and suppression of colon epithelial cell proliferation and regulation of Lgr5+ stem cells leading to prevention of colon cancer (1, 2). This suggests that vitamin D signaling controls gene expression that regulates a diverse spectrum of biological pathways to influence intestinal function. Consistent with this functional diversity, when we applied genomic tools to mouse intestine to investigate the gene targets of vitamin D action across the crypt-villus axis of the small intestine and across the proximal-distal axis of the whole intestine, our data revealed unique spatial patterns of vitamin D action in the intestine that account for compartment-specific functions of this hormone (3).

Our data on the molecular targets of vitamin D action in the intestine extended earlier work by others (7). However very little work on vitamin D mediated gene regulation has been done in normal human intestine. An early microarray experiment using 1,25(OH)_2_D_3_ treated, differentiated cultures of Caco-2 cells found just 12 vitamin D regulated genes, including potential new target genes like amphiregulin, ceruloplasmin, sorcin and Jun b (8). More recent studies identified the 1,25(OH)_2_D_3_-regulated transcriptome in colonoids derived from normal human tissue or colorectal tumors (9, 10). However, these studies do not capture the functional diversity of the intestine.

To understand the impact of 1,25(OH)_2_D_3_ on different cellular compartments (i.e. undifferentiated vs differentiated) and segments (i.e. small intestine vs colon) of the human intestinal epithelium, we used epithelial organoids from human duodenum (enteroids) and proximal colon (colonoids) as experimental models. Enteroids and colonoids derived from intestinal biopsies and maintained as 3-D cultures in an extracellular matrix have emerged as important models to study the development and homeostasis of the human intestine (11). Human enteroids/colonoids can be maintained in a proliferative, undifferentiated-stem cell state or differentiated *in vitro* to make organoids that maintain their regional character (12, 13). Thus, enteroids/colonoids can recapitulate different compartments of the intestinal epithelium as well as the regions from which they are derived. In this study we utilize these models to explore the transcriptional response of different human intestinal segments and compartments to 1,25(OH)_2_D_3_. Our data provide new insight into the molecular actions of vitamin D in the control of human intestinal biology.

## 2 Materials and methods

### 2.1 Establishment and maintenance of human enteroids and colonoids

This study was approved by the Institutional Review Board at the Baylor College of Medicine (under protocol H30712) and informed consent was obtained from all patients. Duodenal enteroids and proximal colon colonoids were established from fresh colonoscopy biopsies (ascending/proximal colon) or resected tissue from roux en y gastric by-pass surgeries (duodenum) obtained from healthy patients through the Texas Medical Center Digestive Diseases Center as previously described (12). Lines were generated from 9 individual subjects with 6 lines examined for duodenum and 6 lines examined for proximal colon (note: 3 subjects had lines developed from both duodenum and proximal colon, **Supplementary Table S1**). Briefly, intestinal tissue was collected and placed into PBS on ice and was washed 3 times. Samples were then placed in a single well of a 6-well plate with Ca^+^ and Mg^+^ free PBS containing 20 mM EDTA at 4°C and shaken for 30 minutes. The tissue was then collected and resuspended in HBSS (with Ca+ and Mg+) and shaken until crypts were released. Crypts were pelleted at 70 x g for 3 minutes at 4°C and the resultant pellet was resuspending in DMEM (containing penicillin-streptomycin and Fungizone) and re-pelleted (70 x g, 1 min). The final crypts were resuspended in Matrigel and plated in a 24 well plate. Following Matrigel solidification, complete medium with growth factors was added (CMGF+; Advanced Dulbecco’s modified Eagle medium (DMEM)/F-12 medium (Thermofisher), supplemented with 50% Wnt3A conditioned medium, (volume/volume), 20% R-Spondin conditioned medium (volume/volume), 10% Noggin-conditioned medium (volume/volume), 100 U/mL penicillin-streptomycin (Fisher Scientific), 10 mM HEPES buffer (Invitrogen), and 1X GlutaMAX (Invitrogen); 1X B-27 supplement (Life Technologies), 1X N-2 supplement (Life Technologies), 1 x B7 supplement, (Life Technologies), 10 mM nicotinamide (Sigma-Aldrich, St. Louis, MO), 1 mM N-acetylcysteine (Sigma-Aldrich), 10 nM Leu-Gastrin I (Sigma-Aldrich), 500 nM A-83 (Tocris Bioscience), 50 ng/mL human EGF (R&D Systems), and 10 nM SB 202190 (Sigma-Aldrich).

Enteroids and colonoids were maintained in CMGF+ with media changes (0.5 ml) every 2–3 days using standard cell culture conditions (37°C, 5% CO_2_, relative humidity 85-95%). Cultures were passaged every week by disrupting the enteroids/organoids with an insulin syringe, followed by resuspension of enteroids/colonoid fragments in Matrigel and replating. Some enteroid and colonoid lines were frozen and stored in liquid nitrogen, and subsequently thawed and expanded again before use.

### 2.2 Experimental protocol for treatment of human colonoids and enteroids

**Figure 1:**
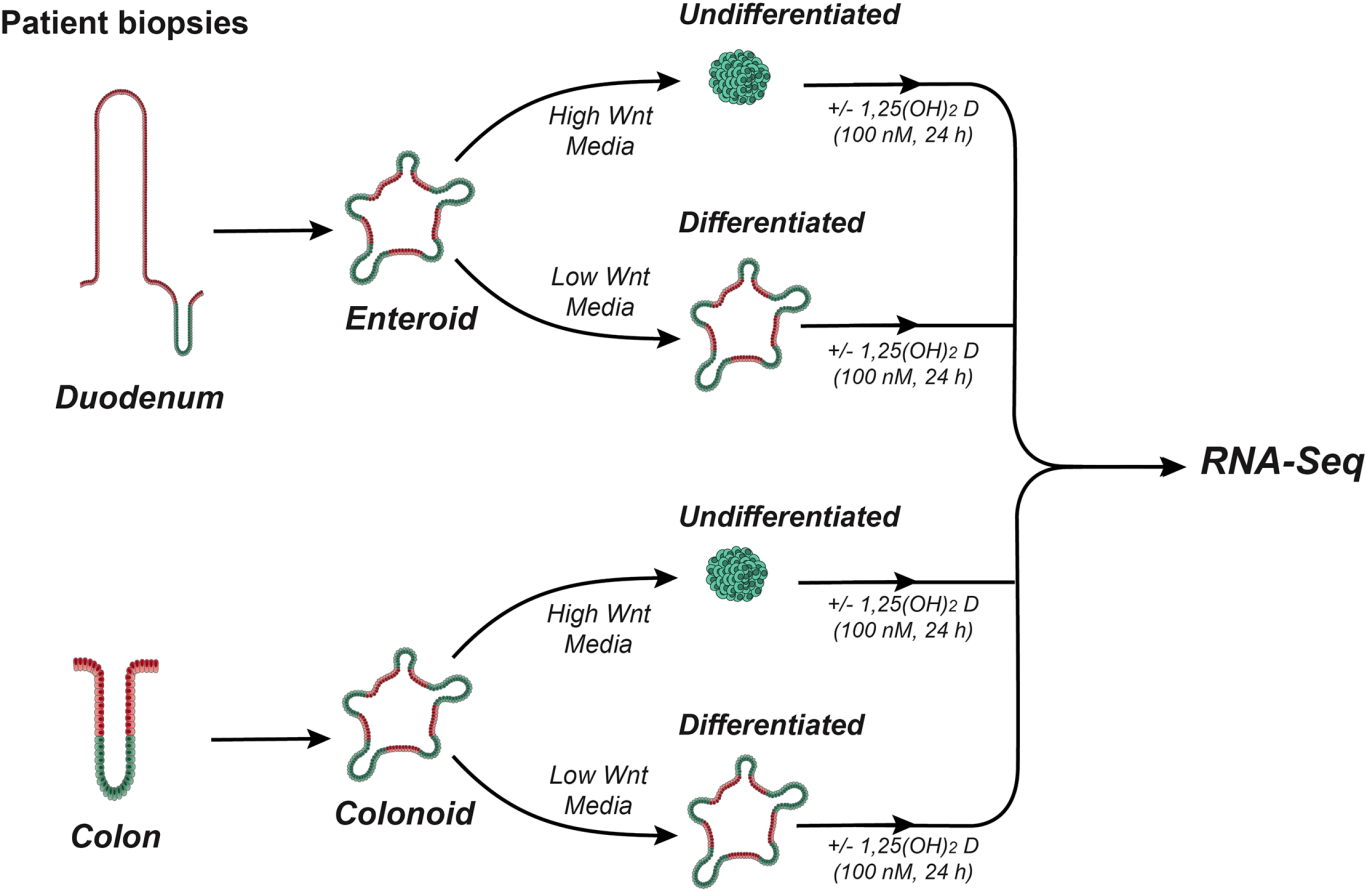
Overview of experimental design. Schematic overview outlining the generation of human duodenal enteroids and colonoids, their treatment to create undifferentiated (stem-cell like) and differentiated (organoid) cultures, and the 1,25(OH)_2_D_3_ treatment protocol.

**Figure 2:**
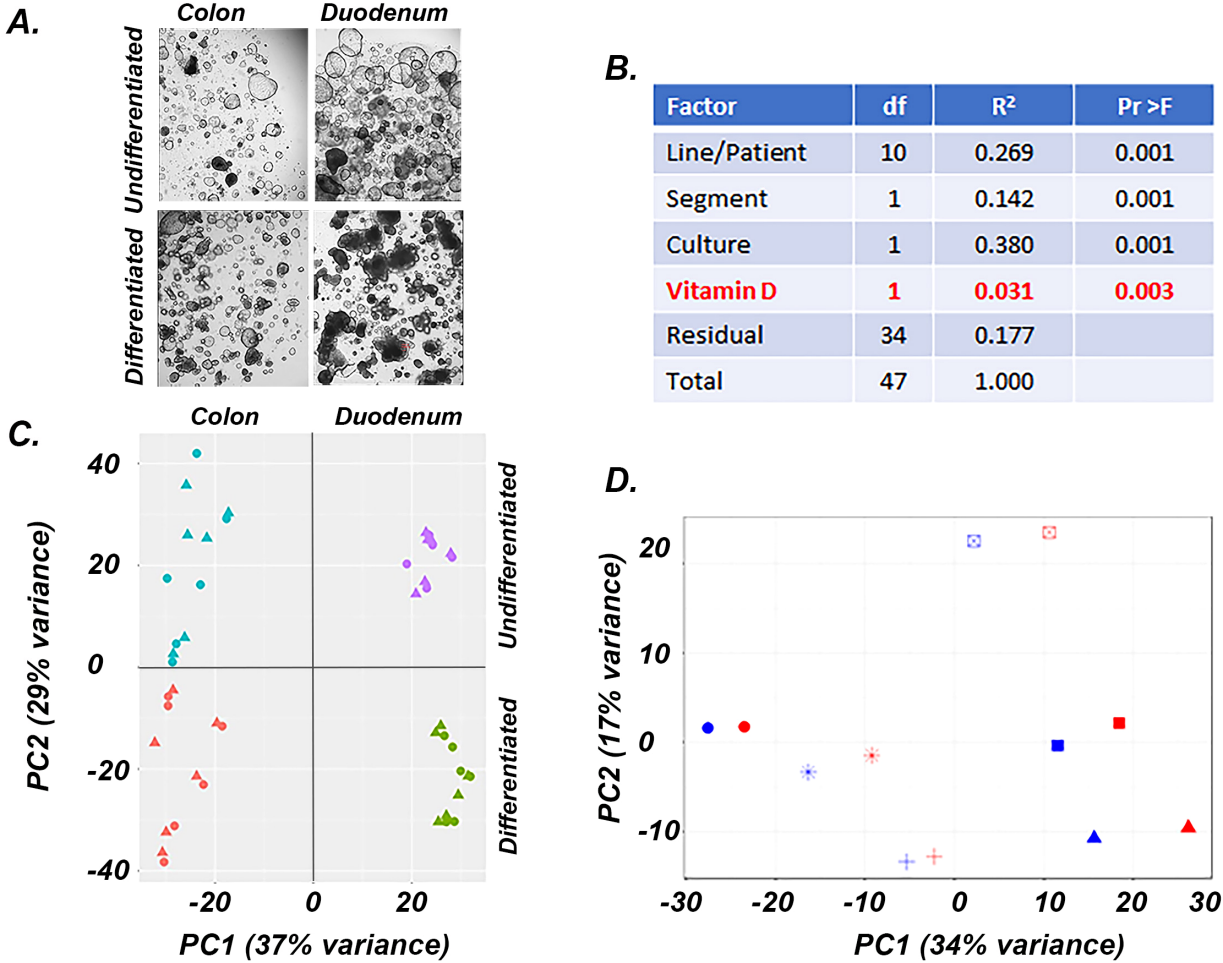
Factors affecting gene expression in human undifferentiated and differentiated colon and duodenal cultures. **(A)** Representative images of the four cultures used for experiments, **(B)** PERMANOVA analysis results showing the impact of various parameters on gene expression, **(C)** PCA analysis of the gene expression data from the four culture conditions, triangles = control; circles = 1,25(OH)_2_D_3_ treated **(D)** PCA analysis of the gene expression data from differentiated duodenal enteroids, each patient sample is shows as a different symbol, blue = vehicle treated, red = 1,25(OH)_2_D_3_ treated.

Six duodenal lines and 6 proximal colon lines were used for this experiment (**Supplementary Table S1**). Enteroids and colonoids were passage into new Matrigel with the goal of having a density of 100 colonoids/enteroids per well. To create undifferentiated cultures, enteroids and colonoids were treated with CMGF+. To generate differentiated enteroids and colonoids, cultures were treated with differentiation medium CMGF- (CMGF+ without Wnt3A, R-Spondin, nicotinamide, or SB202). All cultures were grown for 3 days with one 0.5 ml media change. Subsequently, the cultures were treated with 0.5 ml media containing 100 nM calcitriol (Cayman) or vehicle (ethanol). Three wells were used for each sample and treatment condition and 24 hours following treatment, cultures were collected, pooled by treatment condition, and processed. Calcitriol was prepared from powder to a concentration of 10^−4^ M in ethanol then the calcitriol stock was serially diluted to 100 nM in culture medium. The experimental design is summarized in **Figure 1**. Images of the four types of culture used for study are presented in **Figure 2A**.

### 2.3 RNA Isolation and RNA-sequencing

Total RNA was isolated from differentiated or undifferentiated enteroids/colonoids using the E.Z.N.A.^®^ Total RNA Kit I (Omega BIO-TEK) according to the manufacturer’s protocol. RNA integrity determined using an Agilent 2100 Bioanalyzer. All samples submitted for bulk RNA sequencing had RIN scores greater than 7. Sequencing was performed using paired-end Illumina sequencing by Novogene (Novogene Corporation) following their standard procedures. Subsequently, sequences were aligned to reference genome hg38 using Kallisto (https://pachterlab.github.io/kallisto/) with output expressed as transcripts per million (TPM) (14). Sequencing data was submitted to GEO and is available as accession number GSE159811.

### 2.4 Statistics and bioinformatics analysis

Data was analyzed in R using the DESeq2 package with output expressed as Fragments Per Kilobase of transcript per Million mapped reads (FPKMs). Differential expressed gene (DEG) analysis was conducted with DESeq2 in R using adjustments for the paired nature of our samples. False Discovery Rate (FDR)/adjusted p-values were generated Benjamin-Hochberg procedure that balanced type I and II statistical errors and differential gene expression was calculated at FDR (padj) < 0.05 or 0.1 both with and without a 1.5 fold-change cut off. Multivariate analysis of variance based on distances and permutations was performed using the PERMANOVA package in R using the adonis function in the vegan package with 999 permutations and pairwise distances were calculated using the Euclidean method. The model used included patient origin (Line), the intestinal segment (Segment), the culture condition (Culture), and vitamin D treatment status (Vitamin D).

Functional enrichment analysis was conducted using DAVID (15) and Gene set enrichment analysis (GSEA) (16). In DAVID the analysis was conducted using a background data set of 15,047 genes that were “present” in the human colon or duodenal cultures and for which a gene name/symbol was available. Analyzes were conducted by individual group and separately for up and down regulated transcripts using genes that met the 0.05 FDR cut-off. Analysis was conducted with medium stringency using three gene ontology categories (molecular function, biological process, cell compartment) and three pathway groups (Reactome, Biocarta, Kegg). Categories that were significantly enriched at FDR <0.05 were prioritized for interpretation. When no categories met the FDR cut-off, those with p-values < 0.01 were considered. In GSEA we examined enriched canonical pathways, hallmark pathways, and gene ontology terms using curated gene sets in the Molecular Signatures Database (MSigDB v.2022.1.Hs) (16, 17). The analysis was conducted on 16,770 transcripts identified is “present” in at least one sample set (50% of samples with FPKM values >0.25 for a gene in a group). The dataset was not collapsed, and default parameters were used.

### 2.5 Comparison to other genomic data

We compared the DEG from our RNA-seq analysis (10% FDR cut off) to published RNA-seq data from several studies: (a) Fernandez-Barral et al. (9), 2469 DEGs (1097 up, 1372 down), 1,25(OH)_2_D_3_ (100 nM, 96 h) treatment of undifferentiated colonoid cultures derived from normal colon tissue adjacent to tumors of 6 colorectal cancer patients (GSE100785); (b) Li et al. (10), 1,25(OH)_2_D_3_ (100 nM, 6 or 24 h) treatment of undifferentiated colonoid cultures from a single patient but with 3 technical replicates (GSE100785); (c) Bustamante-Madrid et al. (18), 1,25(OH)_2_D_3_ (100 nM, 6 or 24 h) treatment of undifferentiated colonoids or differentiated colonoids from normal colon tissue adjacent to tumors of 6 colorectal cancer patients (10 ng/g BW, 48h; GSE248274), and (d) Aita et al. (3), 1,25(OH)_2_D_3_-regulated (100 nM, 4 h) expression of genes in small intestinal villus, small intestinal crypts, or colon mucosa isolated from mice (n=6–7 per group; GSE161038). Genomic VDR binding sites used were from vehicle and 1,25(OH)_2_D_3_ treated undifferentiated colonoids (n=182 peaks) (9), LS180 human colon cancer cells (n= 845 peaks), and RWPE1 human prostate epithelial cells (n=7032) (19).

## 3 Results

### 3.1 Factors affecting gene expression in the intestinal cultures

Using Permutational multivariate analysis of variance (PERMANOVA) to identify the drivers of variance among samples (**Figure 2B**) we found that differentiation status (Culture, 37.9%), patient source (Line, 26.9%) and intestinal segment (Segment, 14.2%) accounted for the bulk of the variation in our model. After adjusting for these factors, 1,25(OH)_2_D_3_ treatment had a significant impact on the transcriptome (Vitamin D, 3.1% of the variance). Principal component analysis (PCA) on the entire data set revealed similar results to the PERMANOVA analysis (**Figure 2C**), with 37% of variance separating the duodenal and colonic derived organoids (PC1) and 29% of the variance separating differentiated and undifferentiated samples (PC2). In each culture group, patient line was a stronger driver of variance than 1,25(OH)_2_D_3_ treatment, even though consistent effects of treatment were seen for each line/patient (**Figure 2D**, differentiated duodenal enteroids). Because of the strong impact of line and segment on the transcriptome, we used a paired DESeq2 procedure to control these effects and to identify genes that were differentially expressed by culture condition (i.e. differentiation) and 1,25(OH)_2_D_3_ treatment.

### 3.2 Validation of culture conditions for development of differentiated and undifferentiated enteroids or colonoids

**Figure 3:**
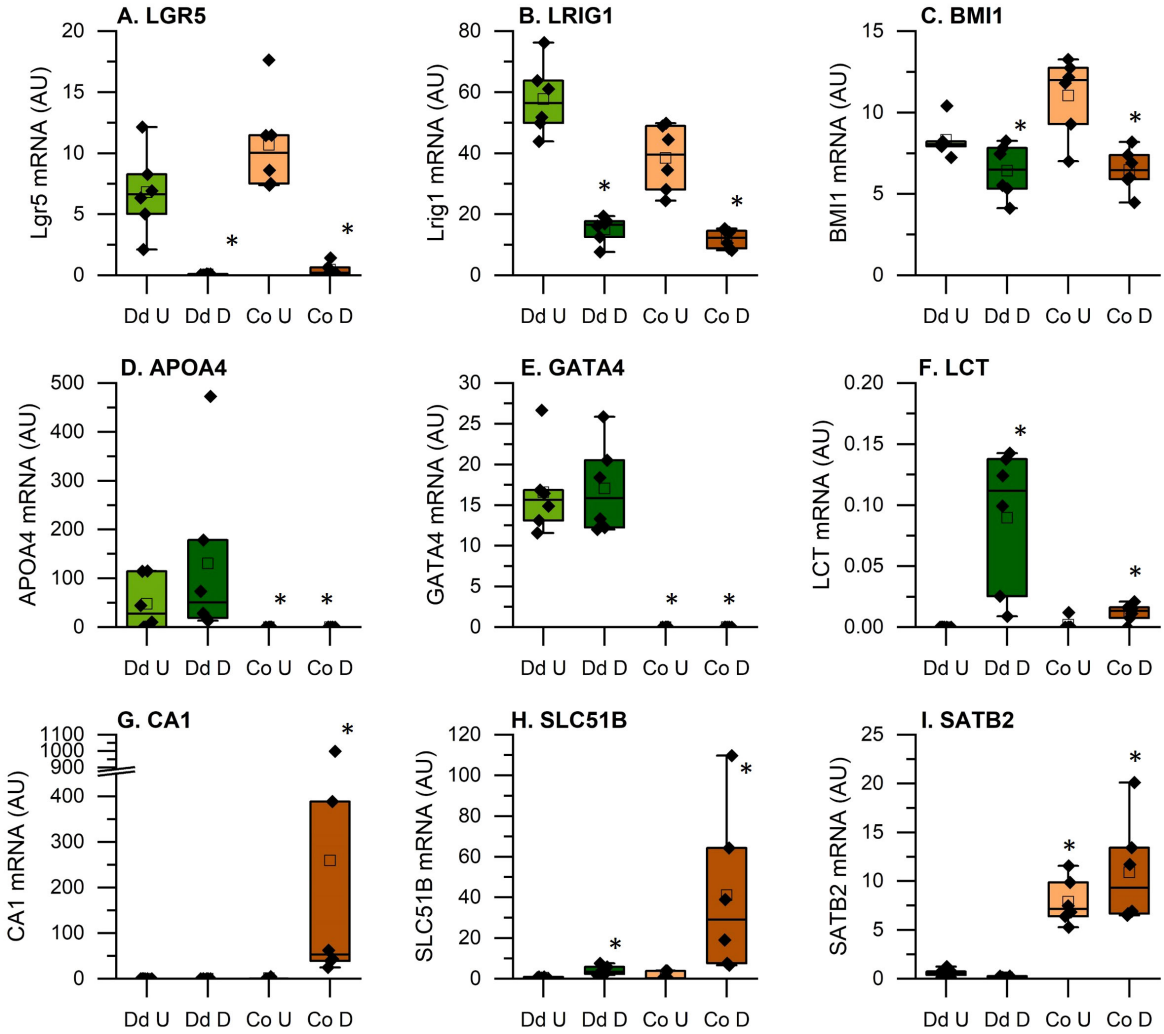
Expression of genes that verify the identity of the tissue origin and the effectiveness of the differentiation protocol. Data are expressed as Fragments Per Kilobase of transcript per Million mapped reads (FPKMs) from duodenal and colon cultures grown under differentiated and undifferentiated conditions. **(A-C)**: Intestinal stem cell markers; **(D-F)**: duodenal epithelial cell markers; **(G-I)**: colon epithelial cell markers *Significantly different from undifferentiated cultures from the same tissue at 5% FDR (n=6 per tissue, differentiation state, and treatment condition).

To demonstrate that the culture conditions achieved the expected goals of modeling both intestinal segment and differentiation state, we examined the RNA-seq data for known markers of various intestinal cell states. The active intestinal stem cell marker *LGR5* was dramatically higher in undifferentiated cultures and two markers for reserve intestinal stem cells, *LRIG1* and *BMI1*, were higher in undifferentiated cultures (**Figures 3A–C**). *APO4* and *GATA4* are markers of the duodenum and were elevated in the duodenal cultures (**Figures 3D, E**) while *LCT* is a marker of the differentiated small intestinal epithelium and was highest in the differentiated duodenal enteroids (**Figure 3F**). Similarly, *SATB2* is a colon cell marker that was elevated in the both colon cultures while *CA1* and *SLC51B* are markers of the differentiated colonic epithelium and were elevated only in the differentiated colonoids (**Figures 3G–I**).

### 3.3 Compartment and cell state influence of 1,25(OH)_2_D_3_ mediated gene regulation

**Figure 4:**
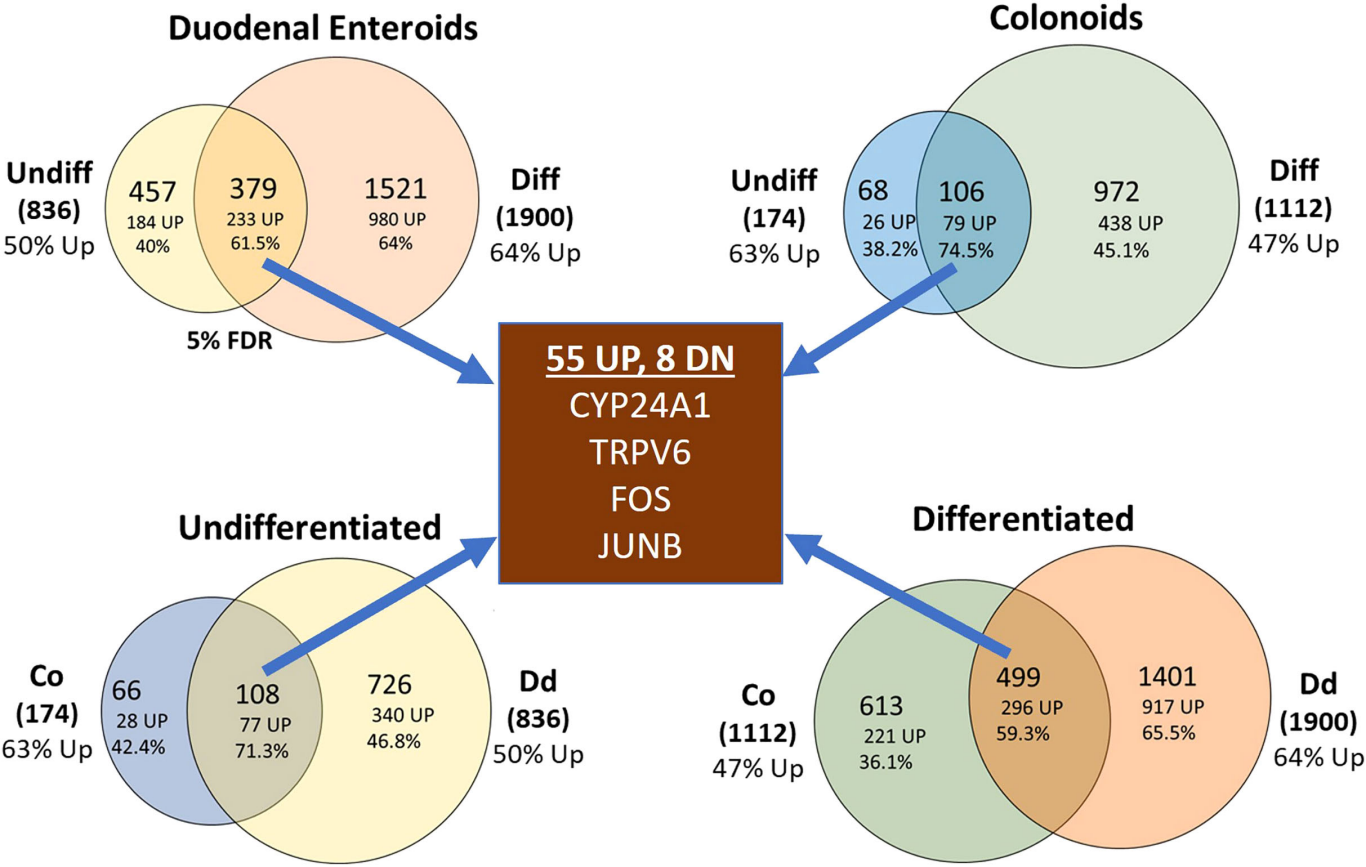
Venn diagrams summarizing the impact of vitamin D treatment on gene expression in the undifferentiated or differentiated duodenal and colon cultures. Top left: duodenal (Dd) cultures; Top right: colon (Co) cultures; bottom left: undifferentiated cultures, bottom right: differentiated cultures. Data represent differentially expressed genes that were expressed at the 5% FDR level. Percentages refer to the upregulated differentially expressed genes.

When examining our RNA-seq data at the 5% FDR cut-off, we identified many differentially expressed genes (**Supplementary Table S2**; see **Supplementary Figure 1** for heat maps of the top 50 up and down regulated transcripts within each of the four culture conditions). A traditional Venn diagram is provided as **Supplementary Figure 2** while **Figure 4** shows pairs of comparisons to emphasize several important points. First, our data show that differentiated cultures have more DEGs in response to 1,25(OH)_2_D_3_ treatment. Undifferentiated enteroids had less than one-half as many differentially expressed genes (DEGs) as differentiated enteroids and undifferentiated colonoids were the least responsive cell type we studied (with only 174 DEGs vs 836 DEGs in the next lowest group). Second, we found that there was a significant overlap in the vitamin D-induced DEGs for all the planned comparisons (i.e. within duodenal enteroids, colonoids, undifferentiated cultures or differentiated cultures) and the overlapping genes were more likely to be upregulated (59.3-74.5%, depending upon the comparison group). Finally, there was a subset of 63 genes that were differentially regulated across all four culture conditions and 87% of these overlapping transcripts were upregulated (**Figure 4**, **Supplementary Table S2**). Collectively these findings confirm a core finding from our previous analysis of the mouse intestine (3), i.e. that there are both common and compartment/segment-specific vitamin D target genes.

**Figure 5:**
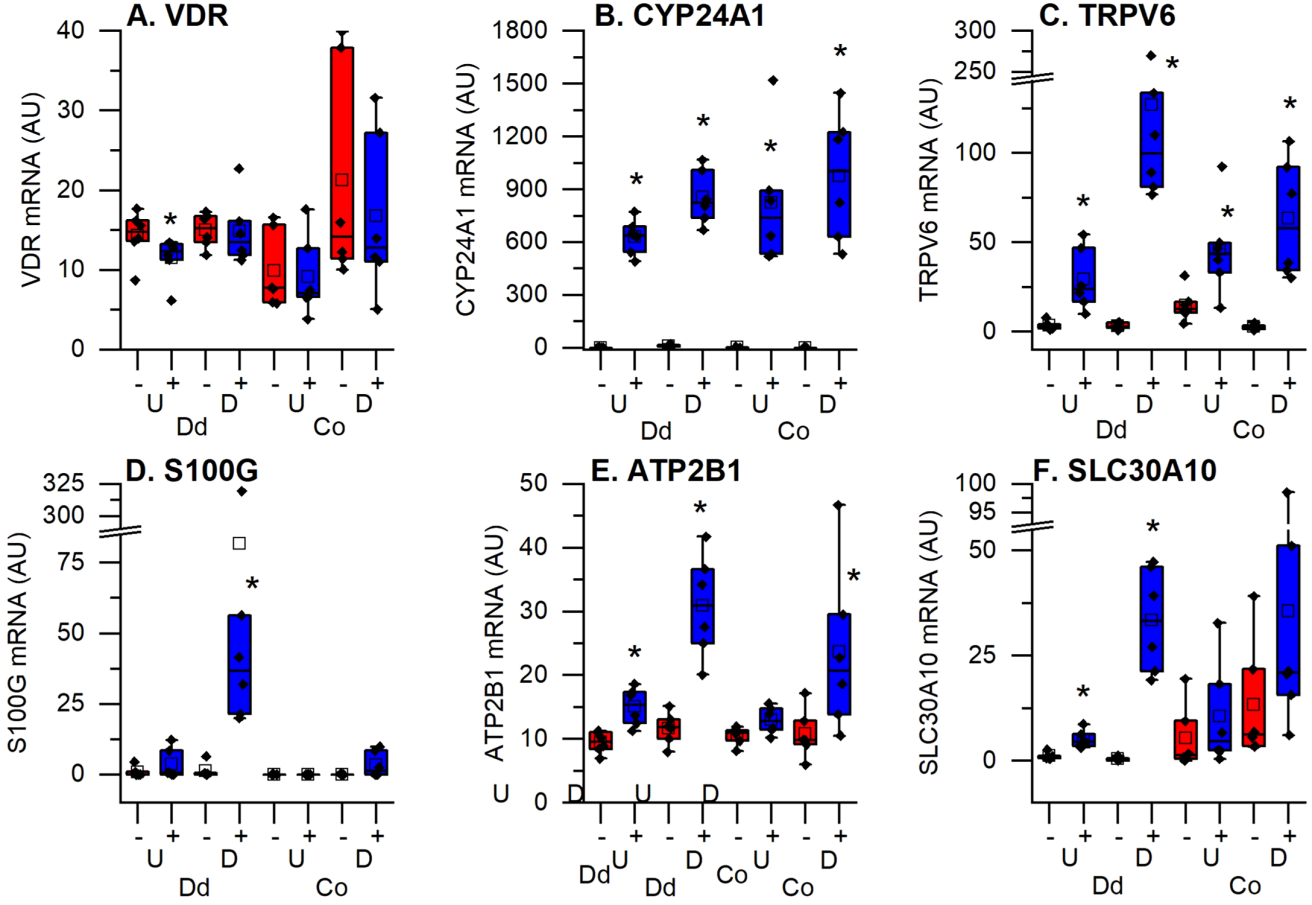
Impact of vitamin D treatment on traditional vitamin D target genes in undifferentiated spheroids (U) or differentiated organoids (D) from the duodenum (Dd) or ascending colon (Co) of humans. **(A)** Vitamin D receptor (VDR); **(B)** 25 hydroxyvitamin D 24 hydroxylase (CYP24A1); **(C)** Transient receptor potential cation channel subfamily V member 6 (TRPV6); **(D)** Calbindin D9k (S100G); **(E)** Plasma membrane calcium channel 2b (ATP2B1); **(F)** Solute carrier family A member 10 (SLC30A10). Data are expressed as Fragments Per Kilobase of transcript per Million mapped reads (FPKMs) from duodenal and colon cultures grown under differentiated and undifferentiated conditions that were treated with vehicle (-) or 1,25(OH)_2_D_3_ (+) for 24 hours. * = differentially expressed by vitamin D treatment at 5% FDR (n=6 per tissue, differentiation state, and treatment condition).

It has been previously established that 1,25(OH)_2_D_3_ binds to the VDR to induce genes encoding proteins necessary for transcellular intestinal calcium transport (i.e. TRPV6, S100G, ATP2B1) and for 1,25(OH)_2_D_3_ catabolism (CYP24A1) (6, 7). **Figure 5A** shows that *VDR* levels were uniformly expressed across the four culture conditions and that 1,25(OH)_2_D_3_ did not alter *VDR* levels; this is consistent with our previous report showing that *VDR* was not induced in small intestinal crypts and villi or in colon mucosal scrapings (20). In contrast, *CYP24A1* was absent in untreated cells but was uniformly induced by 1,25(OH)_2_D_3_ across the four culture conditions (**Figure 5B**). This is consistent with what we have reported in mouse intestine (3). As expected *TRPV6*, *S100G*, *ATP2B1*, and *SLC30A10* were each strongly induced by 1,25(OH)_2_D_3_ in differentiated duodenal enteroids (**Figures 5C–E**). Like *CYP24A1*, *TRPV6* was significantly induced in all four culture conditions. In contrast, *ATP2B1* was not induced in undifferentiated colonoids, *SLC30A10* was induced only in the duodenum, and *S100G* was only significantly induced in the differentiated enteroids. While *S100G* encodes a protein proposed to be involved in intestinal calcium absorption (calbindin D_9k_), other S100 genes encode for calcium binding proteins that regulate cell proliferation/differentiation, apoptosis, metabolism, and inflammation (21). Several S100 mRNAs were also upregulated in differentiated duodenal enteroids (e.g. *S100A2*, *S100A6*, *S100A10*) while S100P, which is associated with tumor growth and colon cancer was downregulated by 1,25(OH)_2_D_3_ treatment in differentiated colonoids (**Supplementary Table S2**).

### 3.4 Functional enrichment analysis reveals vitamin D mediated regulation of many functions and pathways

Enrichment analysis for GO terms and pathway analysis revealed common, as well as segment and differentiation state-specific molecular functions regulated by 1,25(OH)_2_D_3_ (**Table 1** DAVID analysis summary; **Table 2** GSEA analysis summary; Full analyses are available in **Supplementary Tables S3**–**S7**). The diversity of these enriched functions suggests that 1,25(OH)_2_D_3_ regulated additional intestinal functions beyond its traditional role in calcium absorption. Using GSEA, we identified enrichment of cell signature gene sets that suggest vitamin D treatment was reducing cell proliferation and promoting differentiation (e.g. enrichment of gene sets for proliferating cells or stem cells in the Control group for the duodenal cultures; enrichment of mature enterocyte gene sets in the Vitamin D group for undifferentiated duodenum enteroids and colonoids). We also found that in the analysis of differentiated colonoids, the control group was enriched for cell signatures of goblet cells. Consistent with this, the DAVID analysis showed vitamin D-mediated down regulated genes were enriched for the pathways O-glycan processing and mucin biogenesis, two processes typical of goblet cells (e.g. *B3GNT2*, *GALNT7*, *10*, *11*, and *12*, *MUC2*, *MUC6*). This suggests vitamin D treatment antagonizes goblet cell differentiation.

**Table 1:** Summary of Gene Ontologies (GO) and Canonical pathways that are enriched in vitamin D upregulated genes from the four culture groups by NIH DAVID analysis.

Analysis	Duodenum Undifferentiated	Type	Signif *	Duodenum Differentiated	Type	Signif
**Up**	Xenobiotic/Drug metabolism	Reactome	F	Xenobiotic/Drug metabolism/Phase I	GO_ BP, Reactome	F
	Response to wounding	GO_BP	p	Response to wounding	GO_BP	p
	Rho GTPase signaling	GO_BP; Reactome	F	Rho GTPase related	Reactome	F
	Integrin Signaling pathway	Biocarta	p	Regulation of actin cytoskeleton	Kegg	F
	Metabolism/amino acid metabolism	Reactome	F	Cell junction organization/Tight Junction	Reactome/Kegg	F/F
	Lipid/cholesterol/steroid metabolic processes	GO_BP	p	Cell-cell adhesion/communication	GO_ BP/Reactome	F/F
	Translation initiation/elongation	Reactome	F	Cell adhesion molecule/integrin binding	GO_MF/Biocarta	F/p
	Cell response to oxidative stress	GO_BP	p	Response to hypoxia	GO_ BP	F
	Chemical carcinogenesis - ROS	Kegg	F	PI3K-Akt signaling pathway	Kegg	F
	EPH-Ephrin signaling	Reactome	F	TGF beta signaling pathways	Reactome	F
	Cell migration	GO_BP	p	Positive regulation of cell migration	GO_ BP	F
	AP-1 complex	GO_CC	F	Metabolism of Lipids	Reactome	F
	Oxidoreductase activity	GO_MF	F	Cholesterol Biosynthetic	Reactome	F
**Down**	Cyclins during G2/M	Reactome	F	Cyclins and cell cycle regulation	Biocarta	p
	Wnt signaling pathway	Kegg	F	Transmembrane transport	GO_BP	p
	Termination of O-glycan biosynthesis	Reactome	F	Transepithelial water transport	GO_BP	p
	Mineral absorption	Kegg	F	Mineral absorption	Kegg	F
	Transport of cations/anions/amino acids	Reactome	F	Protein digestion/absorption	Kegg	F
	Cell-cell adhesion	GO_BP	p	Metabolic pathways/sulfer metabolism	Kegg	F
	Actin binding/microtubule binding	GO_MF	p	Bile acid synthesis	Reactome	F
	Passive transport by aquaporins	Reactome	F	Fructose metabolism	Reactome	F
				Metabolism of lipids	Reactome	F

Analysis	Colon Undifferentiated	Type	Signif	Colon Differentiated	Type	Signif
**Up**	Drug/xenobiotic catabolic or metabolic process/phase I/cytochrome P450	GO_BP, Kegg, Reactome	F	Xenobiotic/retinoid/estrogen catabolic processes	GO_BP, Reactome	F
	Wound healing	GO_BP	p	Nuclear receptors in lipid metabolism	Biocarta	P
	Chemical carcinogenesis - ROS	Kegg	p	Bile secretion	Kegg	F
	Extracellular matrix organization	Reactome	p	Slc-mediated transport	Reactome	F
	Response to LPS	GO_BP	p	Metabolism/Metabolism of Lipids	Reactome	F
	Oxidoreductase activity	GO_MF	p	Oxidoreductase activity	GO_MF	F
**Down**	Passive transport by aquaporins	Reactome	p	integrin-mediated signaling pathway	GO_BP/Reactome	P/P
	Negative regulation of MAPK pathway	Reactome	p	Cell-cell adhesion	GO_BP	F
	Positive regulation of apoptosis	GO_BP	p	Extracellular matrix organization	GO_BP/Reactome	P/F
				Bile secretion	Kegg	p
				O-glycan processing/Mucin biosynthesis	GO_BP/Kegg	P/P

* F = 10% FDR , P = unadjusted p-value <= 0.01.

**Table 2:** Summary of GSEA analysis of the vitamin D regulated differentially expressed genes using the Gene sets:

Enriched^#^	Duodenum Undifferentiated	Type	Signi *	Duodenum Differentiated	Type	Signif
**Vit D**	Xenobiotics/Phase I functionalization/Cytochrom P450	Reactome	F	Xenobiotics/Phase I functionalization/Cytochrom P450	C5, Reactome	F
	Busslinger duodenal differentiating stem cells	C8	F	Busslinger duodenal mature enterocytes	C8	F
	ILK target genes	C8	F	Glucuronidation	Reactome	F
	Oxidative phorphorylation/Mitochondrial large and small subunit	C5	F	TGF beta Receptor signaling to activate SMADS/Smad heterodimers	Reactome	F
	Eukaryotic translation initiation/elongation	Reactome	F	Rho GTPases activate PKNS/Cycle	Reactome	F
	Ribosome biogenesis/Ribonuclear protein complex	C5	F	Cell extracell matrix interactions	Reactome	F
	Regulation of mitochondrial gene expression	C5	F	Ephrin signaling	Reactome	F
**Control**	Gao Large intestine adult CH MKI67 high cells	C8	F	Gao Large intestine adult CH MKI67 high cells	C8	F
	Gao Large intestine 24W C2 MKP67 pos progenitor	C8		Gao Large intestine 24W C2 MKP67 pos progenitor	C8	F
				Gao Small intestine 24W C3 enterocyte progenitor subtype 1	C8	F

Enriched	Colon Undifferentiated	Type	Signif	Colon Differentiated	Type	Signif
**Vit D**	Bussinger Duodenal mature enterocytes	C8	F	Abnormality of Vitamin D metabolism	C5	F
	GAO Large intestine 24W C1 DCLK1 Pos progenitor	C8	F	Xenobiotics/Phase I functionalization/Cytochrom P450	Reactome	F
				Glucuronidation	Reactome	F
				Rho GTPases activate PKNS	Reactome	F
				Base excision repair	Reactome	F
**Control**	Wnt protein binding	C5	F	GAO_Small intestine 24W C6 Goblet cells	C8	F
	O linked glycosylation	Reactome	F			

^#^ Vit D = pathways enriched in the 1,25(OH)_2_D-treated group; Control = pathways enriched in the vehicle group; * F = 10% FDR.

Our analysis revealed that multiple terms for the functional category of metabolic processes controlling xenobiotic, steroid, or drug metabolism using cytochrome P450s or UDP-glucuronosyltransferases were enriched in all four groups. The genes in these enriched categories include several regulators of 1,25(OH)_2_D_3_ degradation and excretion: *CYP24A1*, which was strongly upregulated in all four groups, *UGT1A1*, which had higher overall expression in duodenal cultures but was induced in all four groups, and *CYP3A4*, which had a larger fold induction in differentiated organoids compared to the undifferentiated organoids. We also found that while some CYP and UGT genes, like *CYP3A5* were up-regulated by 1,25(OH)_2_D_3_ treatment in all groups, others had compartmental specificity (e.g. stronger up-regulation of *CYP2B6* in colon cultures; upregulation of *CYP2C19* or suppression of *UGT2B17* in duodenal cultures; upregulation of *UGT1A3* and *UGT1A5* in differentiated colon and duodenal enteroids.). Collectively, this shows there are unique segmental and compartment responses of CYP and UGT genes in the intestinal epithelium upon 1,25(OH)_2_D_3_ treatment.

DAVID and GSEA analysis identified Rho kinase signaling pathways as being enriched in vitamin D treated differentiated colon and duodenal enteroids (**Tables 1**, **2**). Rho proteins are important molecular regulators of the cytoskeleton, cell morphology (including tight junction dynamics), and cell migration in the intestine (22). The vitamin D upregulated genes within these gene sets/ontologies included regulators of Rho signaling (e.g. *DOCK5*, *ARHGAP25*, *OPHN1*), cytoskeleton (e.g. *ARPC2*, *AKAP12*, *TUBA1A*, FMNL2), and cell adhesion (e.g. ABL1, CDH1). Pathways for integrin signaling and cell adhesion were also enriched in vitamin D induced genes from differentiated duodenal enteroids (e.g*. ICAM1*, *ITGA2* and *A5*, *TIMP2*, *KLK8*).

A pathway for response to wounding was also enriched in vitamin D-upregulated genes that were expressed across all groups, e.g. genes encoding a regulator of notch signaling, PLLP (Plasmollipin); a protease controlling cell growth and migration, KLK6 (Kallikrein-related peptidase 6); and a coagulation regulator that has been implicated as a modulator of intestinal inflammation and barrier function, THBD (Thromboplastin).

Differentiated colon and duodenal enteroids also had vitamin D-mediated enrichment of lipid metabolism genes that included a regulator of intestinal triglyceride synthesis (*DGAT2*), genes involved in fatty acid metabolism (e.g. *ACAA2*, *ELOVL7*, *FADS1*), and those controlling phospholipid metabolism (e.g. *GDPD3*. *PNPLA8*, *GNPAT*). Vitamin D-mediated gene expression in differentiated duodenal enteroids was also enriched for many genes whose proteins are involved in the metabolism of phosphorus (up, *SLC34A2, SLC20A1*), iron (up, *FTH1*; down, *SLC40A1*, *SLC46A1*), copper (down, *ATP7B*), and sodium-coupled transport (down, *SLC9A3*, *SLC5A1*, *SLC6A19*). Vitamin D treatment also reduced genes for water transport in undifferentiated colon and duodenal cultures (e.g. *AQP1, 3*, and *5*).

Other groups have used RNA-seq to examine the impact of 1,25(OH)_2_ D treatment on colon organoids growing under conditions that promote stem cell survival and proliferation (i.e. high Wnt signaling like our CMGF+ media for undifferentiated cultures) (9, 10). Although these studies reported more vitamin D-mediated DEGs than we found in undifferentiated colonoids (5% FDR =174; 10% FDR = 236), the concordance between the studies for these genes was high (124/236 for Li et al.; 168/236 for Fernandez-Barral) with 111/236 matching across all three studies (74 up, 37 down) and 57 of these genes containing a potential VDR binding site including classical (*CYP24A1*, *TRPV6*) and non-classical (*CD1*, *CEBPD*, *CYP3A4*, *CYP3A5*, *FOS*, *JUNB*, and *TXNRD1*) vitamin D target genes (**Supplementary Table S2**). A report by Bustamante-Madrid et al. (18) recently reported that 103 genes were differentially expressed by 1,25(OH)_2_D_3_ (100 nM, 48h) in colonoids induced to differentiate in media lacking Wnt3a and supplemented with BMP4 and the Notch inhibitor dibenzazepine. We found that 69 of those genes were also in our DEG list for differentiated colon organoids (34 up, 35 down) and 22 of these matched genes had a VDR binding peak. Finally, we compared our human organoid data to our previously reported findings on vitamin D-regulated gene expression in mouse small intestine villus, small intestine crypt, and colon mucosal scrapings (3). Of the 1397 vitamin D-regulated DEGs from Aita et al., 438 genes were also identified as DEGs in the human organoids (122 with a VDR binding peak). Direct comparison between mouse intestinal compartments and the human duodenal or colonic cultures is imprecise because the mouse compartments have more cellular complexity than the human intestinal cultures (e.g. the colon mucosal scraping contains more goblet cells and some tissue resident immune cells). Comparisons with similar compartments showed some similarities: small intestine villus to differentiated duodenum, 72 genes matched (24 with VDR peaks); small intestine crypt to undifferentiated duodenal cultures, 116 genes (38 with VDR peaks); colon mucosal scraping to either differentiated or undifferentiated colon cultures, 59 genes (20 with VDR peaks).

## 4 Discussion

1,25(OH)_2_D_3_-mediated signaling through the VDR is known to have a central role in bone and mineral metabolism through its control of both intestinal calcium and phosphate absorption (1). However, vitamin D action has also been reported to have other intestinal functions like suppression of inflammatory bowel disease through effects on intestinal epithelial cells and gut associated immune cells, cancer prevention through actions on colon stem cells, and enhancing intestinal barrier function through regulation of tight junction proteins (2). This indicates that the role of vitamin D signaling in the intestine is broad. Consistent with this, when we examined how 1,25(OH)_2_D_3_ signaling affects gene expression in mouse small intestinal crypts and villi as well the colon mucosa, we found both common and segment/compartment-specific effects (3). Here, we have confirmed the findings from our mouse work by utilizing human enteroids and colonoid cultures treated to either maintain a stem cell phenotype (undifferentiated) or to promote differentiation into organoids. We found both common and distinct 1,25(OH)_2_D_3_-mediated transcriptional responses in these four different cultures, representing unique compartments and segments of the human intestine. Previous studies to assess the effects of 1,25(OH)_2_D_3_ in segments of intestinal epithelium used either mice (3, 7, 23), ex vivo colon organ cultures (24, 25) or undifferentiated human colonoids (9, 10, 26). While these earlier studies revealed important features of vitamin D action, they generally studied only one segment or compartment of the intestine and some of them were underpowered [e.g. Li et al. examined replicate cultures from just one patient (10)]. As such, our study is the first comprehensive transcriptional analysis of how different segments and compartment of human intestinal epithelium respond to 1,25(OH)_2_D_3_.

In this study we found that *VDR* expression was equivalent across the four culture types. This is consistent with our previous report on intestinal epithelial cells isolated from mouse duodenal crypt and villus or colon (20). In addition, we confirmed the induction of several known vitamin D regulated genes in the undifferentiated and differentiated enteroid/colonoid cultures (e.g. **Figure 5** *CYP24A1*, *TRPV6, S100G, ATP2B1, SLC30A10*). *VDR* expression was not significantly altered by 1,25(OH)_2_D_3_ treatment across different segments and compartments. Studies in rats and mice (3, 7, 27) have reported a similarly low or absent induction of intestinal VDR gene expression by 1,25(OH)_2_D_3_, indicating that 1,25(OH)_2_D_3_ regulation of intestinal target genes does not reflect a direct relationship with VDR expression level (28, 29). Thus, compartment or segment-specific differences in vitamin D-mediated gene expression are more likely to relate to diversity in the epigenetic landscape of DNA in a tissue or are mediated through differential recruitment of coactivators by 1,25(OH)_2_D_3_ (4, 30).

**Figure 6:**
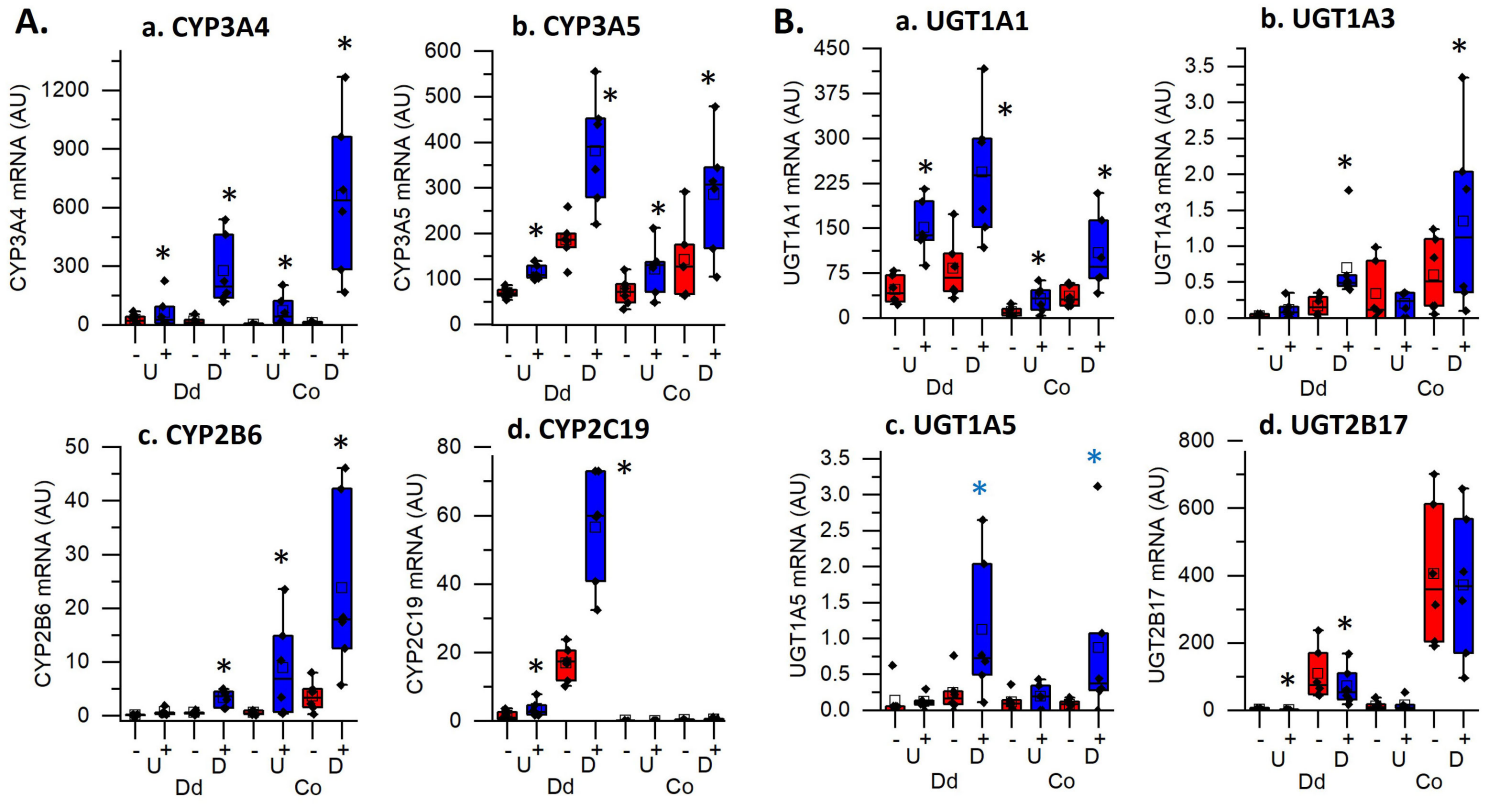
Impact of vitamin D treatment on genes controlling the metabolism of xenobiotics, drugs and steroids in undifferentiated spheroids (U) or differentiated organoids (D) from the duodenum (Dd) or ascending colon (Co) of humans. Data are expressed as normalized Fragments Per Kilobase of transcript per Million mapped reads (FPKMs) from duodenal and colon cultures grown under differentiated and undifferentiated conditions that were treated with vehicle (-) or 1,25(OH)2D3 (+) for 24 hours. **(A)** Cytochrome P450 enzymes; **(a)**. CYP3A4, **(b)**. CYP3A5, **(c)**. CYP2B6, **(d)**. CYP2C19; **(B)** UDP-glucuronosyltransferases (UGTs); **(a)**. UGT1A1, **(b)**. UGT1A3, **(c)**. UGT1A5, **(d)**. UGT2B17. * = differentially expressed by vitamin D treatment at 5% FDR (n=6 per tissue, differentiation state, and treatment condition).

The most consistent process identified as enriched across all four culture conditions in our functional analysis was Xenobiotic/Drug/Steroid Metabolic Processes. This was due to the vitamin D mediated induction of several cytochrome p450s (CYP) and UDP-glucuronosyltransferase (UGT) genes whose protein products both play critical roles in pathways for drug or xenobiotic metabolism and elimination. Although the liver has been viewed as the major organ for xenobiotic metabolism, the intestine also has a major role in xenobiotic detoxification*. CYP3A*4 was vitamin D induced in all four culture conditions with stronger induction in differentiated cultures. It is the predominant CYP family member in the human intestine (31) that contributes to the clearance of the greatest number of xenobiotics and it is also involved in the vitamin D catabolism (32–34). Our observation is consistent with previous studies that noted 1,25(OH)_2_D_3_-mediated, duodenal *CYP3A4* regulation in human colon cancer cells lines (Caco-2, LS180), and undifferentiated human colonoids (8–10) as well as induction of the CYP3A4 homologs *Cyp3a1* or *Cyp3a11* in vitamin D deficient rats, mouse intestinal segments, and in mouse duodenal enteroids (3, 7, 35). In addition to *CYP3A4*, several other CYP mRNAs were also regulated by 1,25(OH)_2_D_3_, e.g. *CYP3A5* in all four cultures, *CYP2B6 i*n colon, and *CYP2C19* in duodenum (**Figure 6**). Because the protein level of these CYPs are lower in human intestine compared to CYP3A4 (CYPA3A4 > CYP2C19 > CYP2B6 > CYP3A5) (36), the functional significance of their induction is less clear. However, CYP2B6 has been reported to mediate the biotransformation of important clinically prescribed drugs (e.g. propofol and methadone) (37) and CYP2C19 plays a significant role in oxidative modification of xenobiotics (38). Homologs of CYP2B6 have previously been reported as being 1,25(OH)_2_D_3_ induced in rat duodenum and in villus from mouse duodenum (3, 35) while 1,25(OH)_2_D_3_ regulation of *CYP3A5* was noted in earlier studies in undifferentiated human colonoids (9).

Vitamin D-induced expression of glucuronosyltransferases (UGTs) suggests that vitamin D may regulate intestinal glucuronidation of lipophilic substrates to increase their water solubility and facilitate their excretion (35, 39). For example, while *UGT1A3* controls hepatic bile and 25(OH)D glucuronidation (40), it is upregulated by 1,25(OH)_2_D_3_ treatment in differentiated duodenal and colonoids suggesting the intestine also has this ability. *UGT2B17* encodes an androgen metabolizing enzyme with higher expression in intestine compared to liver (41); its mRNA was downregulated by 1,25(OH)_2_D_3_ in differentiated enteroids, suggesting vitamin D mediated suppression of *UGF2B17* expression may alter androgenic action in intestine. Consistent with other reports human intestine (38), we found that *UGT1A1* was higher in the duodenum versus colon cultures. In addition, it was upregulated by 1,25(OH)_2_D_3_ in all four culture conditions while others have reported induction rat duodenum and in human undifferentiated colonoids [10; 36]. UGT1A1, plays an important role in metabolizing a range of substrates including steroids, bilirubin, xenobiotics and certain phenols (39) and can protect against irinotecan induced gastrointestinal toxicity (42). Collectively, our findings support a role for 1,25(OH)_2_D_3_-mediated intestinal regulation of human phase I and phase II biotransformation enzymes in the protection against environmental toxins in humans.

Several other functions/ontologies/pathways were also enriched in the RNA-seq data from our intestinal cultures. First, we observed that genes controlling the response to wounding were enriched in each of the four culture conditions (*PLLP*, *KLK6*, *THBD*). This is consistent with a previous report of 1,25(OH)_2_D_3_-mediated upregulation of these three genes in undifferentiated human colonoids (9). KLK6 has been implicated in cutaneous wound healing, proliferation and migration (43), PLLP plays a role in epidermal regeneration and wound healing through its regulatory effects on Notch signaling (44), and THBD has been shown to be important for wound recovery in cultured keratinocytes (45). Although these genes are not well studied in the context of intestinal biology, THBD was recently shown to stimulate the growth of mouse intestinal organoids and to enhance mucosal healing in the murine model of dextran sodium sulfate (DSS)-induced colitis (46). We have previously reported that mice lacking VDR in immune cells delays mucosal healing following DSS-induced colon damage (47) while others have shown that 1,25(OH)_2_D_3_ up-regulates THBD in monocytic cells (48). Future studies will be necessary to determine whether 1,25(OH)_2_D-induced expression of THBD is needed for 1,25(OH)_2_D_3_ mediated healing of the intestinal mucosa in colitis. Related to potential roles for vitamin D signaling in colitis, we saw enrichment in GO terms for the response to lipopolysaccharide (LPS). This includes induction of *FOS*, *JUNB* (i.e. the components of the AP-1 transcriptional complex), and the cell surface glycoprotein, *CD14*, in all four culture conditions as well as the CD14 co-receptor *TLF4* in the colon cultures. Each of these four genes also contain VDR binding peaks suggesting vitamin D signaling mediates both inflammatory and tolerogenic responses to bacteria in the intestinal epithelial barrier (49).

We observed enrichment in several GO terms and pathways related to Rho GTPases affecting cell adhesion, integrins, and cytoskeleton (upregulated genes in differentiated duodenal and colon organoids), and water transport (downregulated genes in undifferentiated cultures). Rho GTPases are a subfamily of G proteins in the RAS superfamily that have an important role in the maintenance of intestine tissue homeostasis (22). This includes regulation of the function and dynamics of intercellular junctions (50) and the control of cytoskeletal plasticity (51). Similarly, the regulation of aquaporins has been associated with effects on intestinal epithelial permeability by controlling water movement across epithelial barriers (52). 1,25(OH)_2_D_3_ has previously been shown to modulate the expression of genes that are involved in the regulation of intestinal tight junction permeability in mice and rats (53–55). Thus, these findings indicate that the 1,25(OH)_2_D_3_ regulation of genes associated intestinal tight junction formation is conserved in humans and further support a role for 1,25(OH)_2_D_3_ as a modulator of intestinal epithelial permeability.

A final category of functional enrichment was GO terms related to lipid metabolic processes and pathways for the metabolism of lipids in differentiated cultures of duodenal and colon organoids. This included genes whose proteins function in a wide range of lipid metabolic processes, e.g. triglyceride synthesis (*DGAT2*), fatty acid uptake *(ELOVL7)*, fatty acid oxidation (*ACAA2*), and unsaturation of fatty acids (*FADS1)*, and phosphatidylcholine metabolism (*ABCB4*, *SLC44A5)*. We previously reported that the intestinal VDR could modulate lipid metabolism in obese mice (56) and lipid metabolism-related pathways were enriched in the vitamin D induced gene expression profile of human prostate epithelial cells (19). We also found that 1,25(OH)_2_ D_3_ treatment caused a small but consistent increase in *CEBPD* in all intestinal cultures and that the *CEPBD* gene has a VDR binding peak in LS180 cells. In liver, CEBPD is an adipogenic transcription factor (57) but its role in intestinal lipid metabolism is not clear. Future studies will be needed to directly test the physiologic importance of vitamin D signaling in the control of intestinal lipid metabolism.

The strength of our research is that it was a comprehensive examination of vitamin D-regulated gene expression in the human intestine. The use of human duodenal and proximal colon cells grown under stem cell-supporting (undifferentiated enteroids/colonoids) or differentiation-inducing conditions (i.e. organoids) thereby provides a broad view of the molecular actions of vitamin signaling in the intestine. In contrast, other studies using human intestinal cultures have focused exclusively on colon and generally emphasize the undifferentiated state (9, 10). Future work should also examine other segments of the small intestine as well as the distal colon and rectum to more fully characterize the molecular actions of vitamin D across the intestinal epithelium. There is some concern that our 24 h time point may be revealing both secondary-regulated as well as primary vitamin D gene targets [i.e. similar to what we previously reported for RWPE1 cells (19)]. However, this problem also affects other published work on colonoids, where treatment times of up to 96 h are common. While additional studies are needed to assess the time and dose dependency of vitamin D-induced gene regulation in human enteroids/colonoids, our 24 h treatment data matches well to this and other published work despite differences in culture conditions, vitamin D dosing duration, and sample size. In contrast, there was poor overlap between the human intestinal culture data and our RNA-seq data from mouse intestinal segments and compartments. This could be due to the early time point we sampled for gene expression in the mouse (i.e. 4 h after treatment vs 24 h continuous treatment in human intestinal cultures), the greater diversity of cell types represented in the mouse tissue (e.g. tissue resident immune cells in colon mucosal scrapings), or potential species differences. In the future we will examine vitamin D-mediated gene expression in mouse intestinal organoid cultures to match the cell populations we studied from the human intestinal cultures. Finally, because we did not generate new VDR ChIP-seq data, we had to rely upon other published VDR binding site data from undifferentiated colonoids or human cancer cell lines (LS180, RWPE1). In addition, while our data suggest that vitamin D signaling is regulating many interesting new genes that control a variety of biological processes, we did not conduct functional studies to demonstrate that the molecular changes lead to a change in the biological function of the intestinal cells. Additional mechanistic studies will be needed to determine the importance of these vitamin D regulated, gene level changes for intestinal biology.

In summary, in this study we used human enteroids/colonoids as models of the human intestinal epithelium and have expanded our understanding of the transcriptional response the human intestine to 1,25(OH)_2_D_3_. Our findings indicate that several genes that have previously been shown to be regulated by 1,25(OH)_2_D_3_ in other models (including mouse models, duodenal biopsies and cancer cells lines) are also regulated in different segments of human intestine. Additionally, we have identified other pathways and genes that can be further explored in future studies to understand how 1,25(OH)_2_D_3_ action may affect phenomena such as intestinal barrier function, first pass drug metabolism, inflammation, and lipid metabolism.

## Data Availability

The datasets presented in this study can be found in online repositories. The names of the repository/repositories and accession number(s) can be found in the article/**Supplementary Material**.

## Supplementary material

Supplementary Figure 1 Heat maps of the top 50 vitamin D- up and down regulated genes in the four experimental groups. Dd, duodenum; Co, colon; Un, undifferentiated cultures; Diff, differentiated cultures.

Supplementary Figure 2 Traditional Venn diagram showing the overlap in vitamin D-regulated differentially expressed genes across the four experimental groups. Dd, duodenum; Co, colon; Un, undifferentiated cultures; Diff, differentiated cultures. Values under the culture names in parentheses are the DEG at 5% FDR and no fold-change cut off. Values within the Venn diagram are the number of DEGs and the percent of the DEG for a culture group that are represented.

Supplementary Table 1 Summary of samples used in the experiments.

Supplementary Table 2 All 3770 genes that are differentially regulated by vitamin D in any of the four organoid cultures at 10% FDR and no fold change cut off.

Supplementary Table 3 Gene Ontologies (GO) that are enriched in vitamin D upregulated genes from the four culture groups from NIH DAVID analysis. BP, biological processes; CC, Cell compartments; MF, molecular functions.

Supplementary Table 4 Canonical pathways that are enriched in vitamin D upregulated genes from the four culture groups from NIH DAVID analysis.

Supplementary Table 5 Gene Ontologies that are enriched in vitamin D downregulated genes from the four culture groups from NIH DAVID analysis. BP, biological processes; CC, Cell compartments; MF, molecular functions.

Supplementary Table 6 Canonical pathways that are enriched in vitamin D downregulated genes from the four culture groups from NIH DAVID analysis.

Supplementary Table 7 GSEA analysis of the vitamin D regulated differentially expressed genes. Gene sets: C3, gene sets for microRNA and transcription factor targets; C5, Gene Ontology (GO) terms for biological processes (BP), Cell compartments (CC), or molecular functions (MF); Reactome, canonical pathways from the Reactome pathway database; C8, cell type signature gene sets.
