# Supplementary figures and images for: Analysis of 1,25-dihydroxyvitamin D genomic action in human enteroids and colonoids reveals multiple regulatory effects of vitamin D in human intestinal physiology
Source: Front Endocrinol (Lausanne). 2025 Jun 4;16:1538463. doi: 10.3389/fendo.2025.1538463 (PMC12173921; doi:10.3389/fendo.2025.1538463)

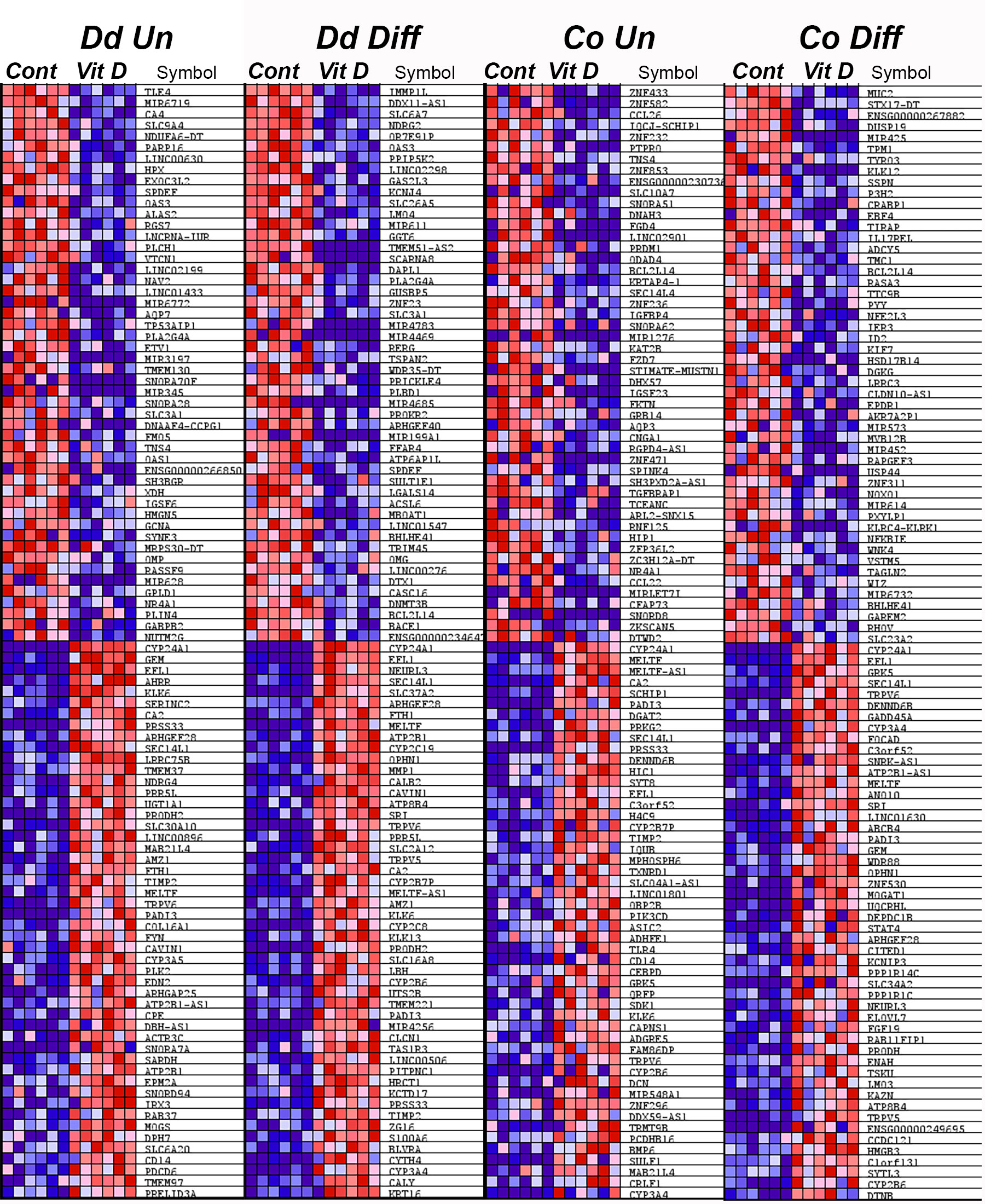

Supplement: Supplementary Figure 1 — Heat maps of the top 50 vitamin D- up and down regulated genes in the four experimental groups. Dd, duodenum; Co, colon; Un, undifferentiated cultures; Diff, differentiated cultures. [file Image1.jpeg]

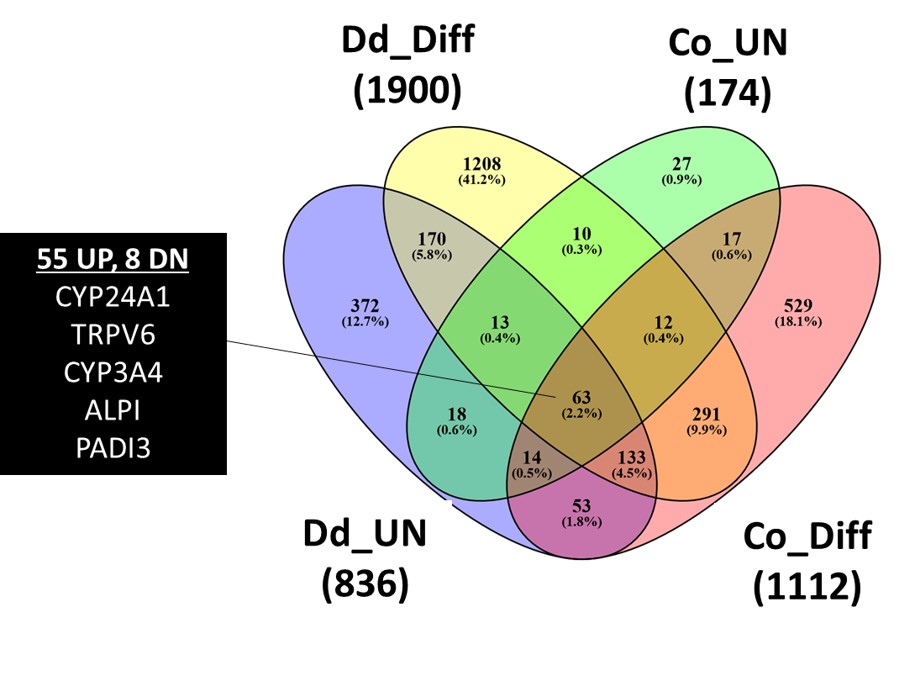

Supplement: Supplementary Figure 2 — Traditional Venn diagram showing the overlap in vitamin D-regulated differentially expressed genes across the four experimental groups. Dd, duodenum; Co, colon; Un, undifferentiated cultures; Diff, differentiated cultures. Values under the culture names in parentheses are the DEG at 5% FDR and no fold-change cut off. Values within the Venn diagram are the number of DEGs and the percent of the DEG for a culture group that are represented. [file Image2.jpeg]
